# Assessment of Adaptive Engagement and Support Model for People With Chronic Health Conditions in Online Health Communities: Combined Content Analysis

**DOI:** 10.2196/17338

**Published:** 2020-07-07

**Authors:** Brian M Green, Katelyn Tente Van Horn, Ketki Gupte, Megan Evans, Sara Hayes, Amrita Bhowmick

**Affiliations:** 1 Health Union, LLC Philadelphia, PA United States; 2 Department of Health Behavior Gillings School of Global Public Health University of North Carolina at Chapel Hill Chapel Hill, NC United States

**Keywords:** social media, social support, health education, qualitative research, patient empowerment

## Abstract

**Background:**

With the pervasiveness of social media, online health communities (OHCs) are an important tool for facilitating information sharing and support among people with chronic health conditions. Importantly, OHCs offer insight into conversations about the lived experiences of people with particular health conditions. Little is known about the aspects of OHCs that are important to maintain safe and productive conversations that support health.

**Objective:**

This study aimed to assess the provision of social support and the role of active moderation in OHCs developed in accordance with and managed by an adaptive engagement model. This study also aimed to identify key elements of the model that are central to the development, maintenance, and adaptation of OHCs for people with chronic health conditions.

**Methods:**

This study used combined content analysis, a mixed methods approach, to analyze sampled Facebook post comments from 6 OHCs to understand how key aspects of the adaptive engagement model facilitate different types of social support. OHCs included in this study are for people living with multiple sclerosis, migraine, irritable bowel syndrome, rheumatoid arthritis, lung cancer, and prostate cancer. An exploratory approach was used in the analysis, and initial codes were grouped into thematic categories and then confirmed through thematic network analysis using the Dedoose qualitative analysis software tool. Thematic categories were compared for similarities and differences for each of the 6 OHCs and by topic discussed.

**Results:**

Data on the reach and engagement of the Facebook posts and the analysis of the sample of 5881 comments demonstrate that people with chronic health conditions want to engage on the web and find value in supporting and sharing their experiences with others. Most comments made in these Facebook posts were expressions of social support for others living with the same health condition (3405/5881, 57.89%). Among the comments with an element of support, those where community members validated the knowledge or experiences of others were most frequent (1587/3405, 46.61%), followed by the expression of empathy and understanding (1089/3405, 31.98%). Even among posts with more factual content, such as insurance coverage issues, user comments still had frequent expressions of support for others (80/213, 37.5%).

**Conclusions:**

The analysis of this OHC adaptive engagement model in action shows that the foundational elements—social support, engagement, and moderation—can effectively be used to provide a rich and dynamic community experience for individuals with chronic health conditions. Social support is demonstrated in a variety of ways, including sharing information or validating information shared by others, expressions of empathy, and sharing encouraging statements with others.

## Introduction

### Overview of the Role of Social Support in Health

Social relationships and social support are purported to promote good physical and mental health for people living with a variety of chronic medical conditions [1-3]. Social support can be defined as “verbal and nonverbal communication between recipients and providers that reduces uncertainty about the situation, the self, the other, or the relationship, and functions to enhance a perception of personal control in one’s experience” [4].

Social relationships and social support can be beneficial to health in many ways. Numerous studies have examined the connection between an individual’s social relationships or networks and their physical health. Research demonstrates that, compared with individuals who are isolated, those who have a social network live longer, experience fewer physical symptoms of illness, and specifically have lower blood pressure [2,5,6]. Poor-quality or low-quality social ties have been associated with the development and progression of certain conditions, including high blood pressure, cancer, cardiovascular disease, autonomic dysregulation, and mortality [2,5,6].

Previous research has also shown an increased likelihood of survival in people who have stronger social ties or social relationships compared with those who have weaker ties or relationships [7]. A review of 81 studies revealed that social support was reliably related to beneficial effects on cardiovascular, endocrine, and immune health [8]. Additionally, social support is associated with increased health-promoting behaviors such as medication adherence [9,10], smoking cessation [11], and weight loss [12,13]. One study in particular showed that providing support was also related to lower measures of systolic and diastolic blood pressure among those who gave support [14].

### Use of the Internet and Social Media for Social Support for Health

The use of the internet has changed how patients, caregivers, friends, family members, and interested citizen-scientists learn about and seek support for specific health conditions. In the United States, 61% of adults search on the web and 39% use social media to find health information [15]. Additionally, 1 in 4 internet users with a chronic illness has gone on the web to find others with similar health problems [16]. It is clear that patients rely on web-based resources, particularly when encountering specific situations, and being diagnosed with a chronic health condition is one such situation.

In a web-based *Health Information Experiences* survey of >2200 patients across 7 health conditions, the authors found that people used web-based resources at some points along their health care journey [17]. For example, 67% of the people used web-based resources when starting a new medication or treatment, 67% when experiencing a new side effect or symptom, 61% when making a medication treatment decision or change, and 57% when needing emotional support [17]. In the same survey, patients were asked about the web-based resources they used; 71% used condition-specific websites and 65% used Facebook for health information and support [17].

Diverse population groups across different races, ethnicities, and education levels access health-related information using social media [18,19]. Social media platforms, such as Facebook, can facilitate dialogue between patients as well as between patients and providers [18].

Using a definition similar to that found in other recent research, online communities are defined in this study as the use of an internet-based platform with the purpose of bringing together a group of individuals with a shared interest or goal [20]. As online communities are hosted on the internet, the geographic location of the member is not a vital component of *membership* in the community or how the community functions. Online support groups and communities have been in existence since the 1990s in various forms. The more recent development of online communities focused on health has been especially beneficial for those living with or caring for someone with a chronic health condition [20,21].

Online health communities (OHCs) are internet-based platforms that allow people to connect over shared experiences and communicate about health [20-22]. OHCs are disrupting traditional hierarchies in the health care industry by offering what traditional health care cannot offer—conversations about the lived experiences of people with particular health conditions. Individuals living with specific health conditions and their caregivers or care partners can browse and search on the web. They can also control the content and flow of information available to them, and, in the case of social media, produce content, rather than just receiving information directly from a health professional in a face-to-face encounter [23]. Although there is criticism surrounding the role of technology in increasing rates of loneliness and social isolation in adults, many view social media as a means to connect with others [24,25].

There are different types of OHCs, both open and closed, with the early versions offering *bulletin board*–style forums where individuals could ask questions and receive responses from other participants. In a closed OHC, a prospective member must opt in to join and be accepted by the site administrators to access site resources and engage with other members, whereas open communities are accessible to all people without having to sign in or provide contact information [20]. Over time, OHCs have evolved to allow for more robust offerings in the ways in which community members can engage through the platform as well as with other community members. OHCs are now more complex ecosystems, capable of being programmed to be better able to adapt and respond to changes in health care and patient experience.

### The Value of Online Communities for Health Promotion

A vital aspect of using OHCs and social media as avenues for health communication is that they can provide valuable peer, social, and emotional support [18,26]. In studies on online communities, 4 types of social support are demonstrated: informational support [27], emotional support [28], instrumental support [29], and appraisal support [28].

An additional type of support that can be garnered by participation in an online community is network support, which is the feeling of being part of a group that shares common interests, which in turn can widen an individual’s social network [29].

Web-based communication and interaction have been shown to provide meaningful support to people seeking health information. This is the case, whether that support is more transactional and provides information support that may help an individual obtain a desired service or make a decision or emotional support where the individual receives validation of their feelings or experiences [30]. Previous research has demonstrated that social support is important for an array of health outcomes and can be provided in various ways. There is evidence that social support can flourish when provided specifically through a web-based environment and can even empower individuals [27,31,32].

Social media offers additional space for dialogue around health information and experiences. Although many individuals find comfort in openly sharing aspects of their health experiences on the web, other people are more passive in how they participate in online communities. Research has identified 2 main types of participation in online communities: active participation, which includes posting and sharing, and passive participation, defined as browsing and reading [33]. Whether participants in an online community are more active or passive, both groups can find the level of support they are seeking [34].

Social support through a web-based medium can offer similar depth, breadth, and level of intimacy that occurs in private offline conversations [21,35]. Face-to-face social support may not always be available or may be difficult for people who have a chronic health condition or are disabled; therefore, OHCs can provide the vehicle through which ongoing and regular interaction is available [29].

However, the effects of web-based social support on chronic disease management are less clear from the available literature [26]. Research examining online cancer support groups found that participants in these groups had decreased levels of depression as well as decreased reactions to pain [36]. Previous research has also examined perceived empathy as a key factor in patient health outcomes [37]. Recent research has shown that users of an online diabetes management community have higher levels of diabetes self-care and health-related quality of life [38]. Despite the value of online communities for engaging people in a variety of health issues, research on the use of social media platforms, such as Facebook, to help people manage chronic health conditions is in its relative infancy [39].

### Purpose of This Study

This study aimed to assess the provision of social support and the role of moderation in OHCs developed in accordance with and managed by the adaptive engagement model described herein and created by Health Union. Health Union cultivates OHCs that are designed to have a positive impact on the lives of people living with chronic conditions by providing the information and support they are seeking. Health Union currently has 27 active condition-specific OHCs and corresponding Facebook pages that serve to extend the reach of the adaptive engagement model.

In this study, the authors, who also serve as OHC community managers, first demonstrate how the adaptive engagement model works to maintain safe web-based spaces for the provision of social support for people with chronic health conditions. In the study model, moderation includes responding to comments; thanking and welcoming comments; providing validation; linking to resources; and maintaining a respectful, inclusive tone. In this paper, the authors elucidate key elements of the model that are central to the development, maintenance, and adaptation of OHCs for people with chronic health conditions. For this study, the model was adapted using the principles of sharing content and supporting engagement on dedicated public Facebook pages to demonstrate the utility of the OHC model.

The Health Union OHC model encourages people to take an active role in their health by providing content that aligns with their needs and interests and by cultivating a safe environment where communication, understanding, and meaningful relationships can thrive. This original content can be accessed on condition-specific websites and their corresponding social media pages. In this study, we refer to this model as the “Health Union Online Health Community Adaptive Engagement Model,” and in previous publications have referred to this as the “meeting people where they are at” model [40].

The OHC adaptive engagement model is developed with the belief that OHCs can be defined and have discernible features. First, similar to in-person communities, OHCs are situational, contextual, and involve a community of choice. There are 3 structural elements of the OHC adaptive engagement model: social support, adaptive engagement, and active moderation. Some features of OHCs can be categorized into more than one of the structural elements as there is often an overlap. These features include the following:

1. Social identity: OHCs, by definition, are for people with a health condition, caregivers, or others who have an interest in the health condition. People who live with a given chronic health condition may choose to identify as a person living with the condition or not, but regardless, being part of an OHC confers a social identity.
2. Social relationships: Relationships with others in OHCs are social relationships. This means they are imbued with a range of characteristics, including reciprocity, mutual support, and emotional connection, with the potential for shared meaning and conflict [41].
3. Interdependence: Being part of a social group means that there is a connection or relationship with others. A relationship entails interdependence in terms of communication, expectations, behaviors, and other aspects of the relationship [41].
4. Experience of societal stigma: Chronic health conditions, because of their very definition as a state of ill health and the fear of contagion that underlies notions of illness in our society, bear a level of social stigma. Some health conditions bear more stigma than others, and individuals living with these health conditions may feel highly stigmatized if there are visible markers of their health condition or when their diagnosis is known to others [42,43]. The experience of stigma and people sharing how they confront or cope with it is an important aspect of community bonding.
5. Boundary marking: The community is defined by the diagnosis or experience of a health condition. Although there are usually clear definitions and an understanding of who is “living with the health condition,” these are based on self-disclosure. In addition, these boundaries are porous and let in new members [44].
6. Social norms: Norms that define interactions of the larger society are represented within OHCs. Often, these social norms are communicated in the form of “community rules,” and OHC participants may need reminders of the rules from time to time. There may be formal (warning and banning from the group) and informal (encouragement to use respectful language) sanctions for those who violate these community rules.

## Methods

### Overview of Methods

The Health Union OHC adaptive engagement model was developed to connect people with information that meets their health needs and as a means of providing social support [32,45]. This model includes a paid community management team encompassing dedicated community leads as well as peer moderators and content contributors who are living with a chronic health condition, caregivers, or health care professionals. Community managers also serve as moderators and may contribute to content [46].

OHCs included in this study are Health Union online communities created for people living with multiple sclerosis, migraine, irritable bowel syndrome, rheumatoid arthritis, lung cancer, and prostate cancer. Each OHC has a website with a dedicated URL and corresponding linked social media channels, including a condition-specific public Facebook page. These OHCs were launched in the following order: Migraine, December 2010 [47]; Multiple Sclerosis, March 2013 [48]; Rheumatoid Arthritis, July 2013 [49]; Irritable Bowel Syndrome, June 2016 [50]; Lung Cancer, January 2017 [51]; and Prostate Cancer, December 2017 [52].

Original content is published on each of the community websites daily and is shared via the corresponding social media pages, including Facebook. All Facebook posts include a link to the original article that was first published on the corresponding community website. These Facebook posts include a customized clickable image with a short engaging message (approximately 80 characters, hereafter referred to as Facebook post copy) that links to an article published on the condition-specific community website. For this study, comments on Facebook posts were the primary unit of analysis. Any comments that were made directly on the condition-specific websites were excluded from this analysis.

Facebook analytics data were used to determine the reach and engagement of the 6 OHCs. The mixed methods combined content analysis (CCA) [53] was used to analyze the comments on 39 sampled Facebook posts from the 6 OHCs identified above to identify themes that aided the authors in illustrating how key aspects of the adaptive engagement model facilitate the different types of social support and other constructs of the model. Additionally, this analysis examined how the adaptive nature of this model can be used to support people regardless of their chronic health condition.

### Facebook Reach and Engagement Data

Reach and engagement data were calculated using Facebook’s analytic tool, Facebook Insights. In this study, the authors used the Facebook definition of engagement as users reacting to (ie, clicking a reaction button such as “like”), sharing, commenting, or clicking on any posted content; in this case, a link to an original article on the corresponding community website. Reach refers to the number of people who saw the Facebook post, image, and short copy either in their newsfeed or by directly visiting the Facebook page. The reported gender and age cohort of those who reached and engaged were also collected. Weekly averages of reach and engagement were calculated for posts spanning the period from January 1 to June 30, 2018 (Table 1).

**Table 1:** Average weekly organic reach and engaged users per Facebook community (Facebook insights data from RheumatoidArthritis.net, ProstateCancer.net, MultipleSclerosis.net, Migraine.com, LungCancer.net, IrritableBowelSyndrome.net Pages, January 1 to June 30, 2018).

Facebook insights data		Rheumatoid arthritis		Prostate cancer		Multiple sclerosis		Migraine		Lung cancer		Irritable bowel syndrome	
Weekly reach^a^ (unique users), n		104,874		6617		160,906		161,958		9328		11,692	
Engaged^b^, n (%)		9176 (8.75)		484 (7.31)		12486 (7.76)		11791 (7.28)		561 (6.02)		1073 (9.18)	
**Gender^c^, n (%)**													
	Female		7800 (85.00)		131 (27.1)		10613 (84.99)		10730 (91.00)		471 (83.9)		987 (91.98)
	Male		1293 (14.09)		353 (72.9)		1786 (14.30)		987 (8.37)		86 (15.3)		78 (7.23)
**Age cohort^c^ (years), n (%)**													
	18-34		403 (4.39)		9 (1.9)		1024 (8.20)		2052 (17.40)		18 (3.2)		95 (8.85)
	35-44		890 (9.69)		15 (3.1)		1872 (14.99)		2712 (23.00)		26 (4.6)		129 (12.02)
	45-54		2019 (22.00)		48 (9.9)		2997 (24.00)		2948 (25.00)		56 (9.9)		182 (16.96)
	55-64		3028 (32.99)		136 (28.1)		3371 (26.99)		2122 (17.99)		180 (32.1)		278 (26.00)
	>65		2753 (30.00)		266 (54.9)		3122 (25.00)		1651 (14.00)		275 (49.0)		365 (34.02)
Total Facebook followers^d^, n		115,232		8579		125,250		170,219		19,461		25,059	

^a^Facebook Insights data, average weekly organic reach (unique users).

^b^Facebook Insights data, average weekly engaged users as the proportion of weekly organic reach.

^c^Gender and age for Facebook-defined engaged users; data reported from user profiles.

^d^Facebook Insights data; total number of followers as of June 30, 2018.

### Sampling and Data Collection

A diverse range of Facebook posts linking to original content from the 6 OHCs was purposefully sampled, resulting in a sample of 39 Facebook posts and their respective comment threads for a total sample of 5881 comments. Each selected post received comments from community members on Facebook. The content of the posts and comments included a broad range of topics, including impact on relationships, coping, quality of life, and treatment experience, all of which comprise the patient journey for people with a chronic health condition.

Using the principles of CCA, it is important to distinguish between the sampling unit, contextual unit, and coding unit as part of the methodology and the resultant analysis [53]. In this study, the Facebook posts with their corresponding extracted comments are the *sampling units*; the Facebook post copy along with the linked article, comments, and/or comment threads are the *contextual units*; and a sentence or phrase within an individual comment (as comments can range from a single word or phrase to a paragraph) are the *coding units*.

A purposeful sampling of the original articles and corresponding Facebook posts was drawn based on the following criteria: a range of content reflecting shared experience and sense of belonging of community members, stigma, and other challenges of living with a chronic health condition. An additional selection criterion for purposeful sampling was that the post needed to have a minimum of 20 comments to be included in the sample.

The Facebook posts that were analyzed for this study (Multimedia Appendix 1) [54-92] were originally posted on one of the OHCs’ Facebook pages between February 11, 2017, and June 5, 2018. The comments from these Facebook posts were extracted into a spreadsheet and imported into a qualitative and mixed method research data web application, Dedoose (Version 8.0.35; SocioCultural Research Consultants, LLC) [93]. Comment transcripts were labeled by community website and Facebook post ID (eg, PC 6 is used to label a specific Facebook post about PC on a specific date/time and all comments associated with that post). A total sample of 5881 comments were distributed as follows: PC, 1576; RA, 1417; migraine, 876; LC, 735; MS, 701; and IBS, 576.

### Combined Content Analysis of Facebook Comments

CCA is a mixed methods approach used to analyze data from social media platforms [53]. This approach allows for the combination of qualitative and quantitative methods, inductive and deductive coding procedures, and manual and automated analytic modes [53]. As stated above, the authors used CCA as a methodological approach for deriving and analyzing a sample of Facebook post comments.

Virtual ethnography methods were used to develop a coding schema using an iterative deductive process [94]. As the authors serve as community managers and moderators, they are immersed in these communities on a day-to-day basis and have the ability to understand and tease apart some nuances in these conversations.

The analysis was carried out in 3 stages. In the first stage, the authors used a randomly selected subset of the sampled comments to identify initial thematic codes using content analysis. Using a concept-mapping process aided by Dedoose, the authors developed a conceptual framework to organize and further analyze the data to achieve theoretical saturation of themes rather than generalizability.

These initial coding samples were each tested by pairing the authors to confirm the interrater reliability (IRR) of the coding schema. The IRR of initial coding was calculated using Pearson correlation coefficient, and coding testing was repeated until the IRR achieved 90% agreement.

The second stage consisted of an in-depth review of these codes by the team, combining and collapsing codes when appropriate and developing the final coding schema with examples to generate the full codebook (Multimedia Appendix 2).

In the final round of coding, the full sample of Facebook post comments was coded by the authors, and an analytical set of thematic categories was identified throughout the full data set using Dedoose. As stated above, comments can be of variable length, from a phrase to several sentences. Code applications to a comment were not mutually exclusive (eg, a given comment could have multiple codes applied). The final set of codes was then juxtaposed against the thematic categories in the analysis to determine their relationship with the structural elements of the OHC model.

The qualitative analysis of the coded comments and patterns of code distributions were used to identify thematic categories that were further compared for similarity and differences across each of the 6 OHCs and by content descriptive category (eg, a comment about symptoms or treatment).

## Results

### Facebook Reach and Engagement

Facebook Insights data, which show the average organic reach (unique users) and engaged users per week for each of these OHCs, is presented in Table 1. Overall, the 6 OHCs reached an average of 455,375 people per week and engaged approximately 6% to 9% of them, which is considered a high level of engagement on a Facebook post [95]. Individual OHCs reached a range of users: from 6617 people in the PC OHC to 161,958 in the migraine OHC. The gender and age cohort of engaged users, as self-reported in individual user Facebook profiles, is presented to show the demographics of the participants. Except for the PC OHC, females made up a large majority of OHC users.

### Facebook Post Comments and Model Elements

Qualitative thematic network analysis of comments from the OHCs corresponds to the structural elements of the Health Union OHC adaptive engagement model—social support, adaptive engagement, and active moderation. Examples of comments that typify each of the structural elements of the model as well as the frequency with which each theme occurred in the comments are presented in Table 2. Overall, the majority (3405/5881, 57.89%) of the comments included one or more codes that represent an element of social support. In addition, almost one-third (1758/5881, 29.89%) of the comments included codes that represent adaptive engagement, and 12.21% (718/5881) reflected an element of active moderation.

Social support was demonstrated in a variety of ways, including giving advice, providing encouragement, and expressing empathy. Sharing knowledge and experience is one such manner of conveying support, with one commenter stating, “There’s many days when these posts remind me I’m not the only one having to deal with these same issues.” This is especially important as people suffering from chronic illness often feel that there are few people who truly understand what they are going through. For example, the previous commenter went on to post:

I constantly get looks and attitudes of ‘you look too young to be so sick, you don’t look sick.’ Even from naïve Dr’s [sic] on occasion and often have people attempt to tell me such. It’s infuriating.

Having a shared experience helps community members to feel understood and less alone, with another commenter stating:

Unfortunately, what I have learned is that unless you are someone who struggles with it, the rest of the world (including some family and health professionals) just doesn’t care. There will be no support nor empathy. Hence why I am thankful for this site.

Community participants share encouragement and express their willingness to be there for each other through hard times. The simple existence of the community, the daily content posted, and the experiences and support shared by users can help those who suffer from chronic illnesses feel less alone. This is shown by comments such as this one:

Your articles seem to come out at the perfect time and speak right to me. It’s as if I unknowingly wrote them myself. Thank you for being the biggest support system I have outside of my family.

Effective moderation by community managers helps to keep the discussion respectful and can help ameliorate some of the harmful effects that are often encountered on the internet, such as misleading medical advice, conspiracy theories, and the like.

**Table 2:** Exemplary quotes demonstrating qualitative themes and their frequency distribution.

Thematic primary code					
Subcode		Exemplary quote		Frequency distribution, n (%)	
**Social support (n=3405)**					
	Empathy and understanding		“I’m glad this post came up. Most people who have never had a Migraine cannot understand the degree of pain and the strange feeling.”		1089 (31.98)
	Knowledge and shared experience from community		“Sometimes I see some of your posts and I think YAY it’s not just me…and your column makes me feel like it is not only just me, but it’s me and 5 million of my closes [sic] friends.”		1587 (46.61)
	Express stigma		“A lot of people don’t think I have a “real” illness, and that includes healthcare professionals. It is probably the most frustrating part of this illness…sometimes I am treated like a drug-seeker, and sometimes I am treated like a nutcase…The only battle worse that getting healthcare professionals to accept my illness and treat it, is getting my family members to accept and understand it as real, and not blame me for it.”		381 (11.19)
	Giving advice		“The monster is never very far away. Write a journal, buy a dog, start doing your bucket list. The busier you stay the better.”		283 (8.31)
	Advocacy and awareness		“It has helped me research, it has ‘pushed out’ things that I need to learn, it has provided a community that I can engage with and be a part of. It has saved me from feeling so alone.”		133 (3.91)
	Sense of belonging/group membership		“I do get much comfort coming here knowing I am not alone in this world.”		378 (11.10)
	Encouragement and motivation		“I applaud your courage and determination to have stayed the course… I plan to stay the course as well.”		579 (17.00)
	Caregiver perspective		“I am caregiver for my husband who is in advance stages. It is becoming more apparent that he will lose the battle due to respiratory issues…Reading your entries remind me that others have gone before me as caregivers for this disease that sucks.”		180 (5.28)
**Adaptive engagement (n=1758)**					
	General (tag person, agreement, and emoji)		“[tagged person] I’m glad this post came up…When you have the time, please read some of what people say in this post.”		432 (24.57)
	Information seeking		“My question is with 15-16 migraines a month, if not more, how [do] you work through them… Any tips would be greatly appreciated!”		332 (18.88)
	Information giving		“I have learned there’s new technology they do in FL and NY called Sperling Prostate Cancer Treatment Laser…”		272 (15.47)
	Conflicts/difference of opinion		“I’m sorry, but I think your outlook is incorrect. You should not avoid asking an important question in order to spare someone’s feelings. The goal should be to remove the negativity toward people that do/did smoke, instead of simply not asking the question.”		51 (2.90)
	Community norms and rules		“Thank you for voicing your concern…I did want to take a moment to clarify that migraine.com is not owned by a pharmaceutical company. You can visit the site for further information about who we are and what we do: https://health-union.com/.”		49 (2.79)
	Boundary marking		“Hopefully you’ll be able to enjoy some support and kinship here, with lots of folks who have similar experiences and can relate to what you’ve said.”		51 (2.90)
**Active moderation (n=718)**					
	Empathy and understanding		“I value you and am grateful to have you as part of our community…we can all relate to one another…and it is comforting to hear a kind response from you and others.”		432 (60.2)
	Share knowledge and resources		“When you have the time, please read some of what people say in this post. I hope the stories help to make those people without migraines understand that it is different for all people and … try to understand odd behavior and debilitating pain.”		298 (41.5)
	Share patient/caregiver experiences		“I am so sorry to hear about the mental health struggles you have alongside your migraine. As we speak, I am 1-2 hours into a sudden and dramatic downturn in mood, and it has me worried about a potential bad migraine.”		207 (28.8)
	Resolve conflicts		“It’s never our intention to make anyone feel that they are difficult to love- I believe the article was trying to address how difficult chronic pain can be in the relationship dynamic, but I hear you, and apologize that was the message that came across. Thanks for your feedback and for being part of our community.”		25 (3.5)
	Maintain community rules		“Since the community is bound to disagree at some points when discussing politics, we wanted to post a friendly reminder that posts of all opinions are welcome as long as they are respectful of each other and not inflammatory.”		24 (3.3)

As shown in Table 2, social support is most frequently demonstrated by sharing knowledge of their health condition and experience among community members (1587/3405, 46.61%) and expressing empathy and understanding (1089/3405, 31.98%). Conversely, very rarely do members provide social support by giving advice (283/3405, 8.31%). Most likely, this is because the OHC community rules explicitly state that we do not provide medical advice [96]. Community managers mirror the behavior of discouraging giving advice and reminding participants that people may use a range of treatment options and have different treatment experiences.

From the perspective of the content featured in the Facebook posts and their respective linked articles, the topics discussed in the comments were a broad range. They included caregiver experiences, complications and comorbidities, complementary therapies, coping with the health condition, diagnosis, emotional impact, experiences with health care provider(s), insurance coverage or disability issues, laboratory and other tests, life impact of chronic illness, lifestyle measures, mental health, patient journey, relationship issues, sexual performance and sexual health, and treatment or specific medication discussions (Table 3).

**Table 3:** Frequency distribution of model elements by topic discussed.

Topic discussed	Number of comments per topic, N	Social support comments, n (%)	Adaptive engagement comments, n (%)	Active moderation comments, n (%)
Caregiver experience	128	102 (79.6)	18 (13.8)	8 (6.9)
Complications and comorbidities	265	173 (65.3)	81 (30.7)	11 (4.0)
Complimentary alternative medicine	142	78 (55.0)	50 (35.0)	14 (10.0)
Coping with condition	358	275 (76.8)	60 (16.8)	23 (6.4)
Diagnosis	181	125 (69.1)	32 (17.7)	6 (3.1)
Emotional impact	425	282 (66.4)	102 (24.0)	41 (9.6)
Health care provider experience	295	139 (47.1)	111 (37.5)	45 (15.3)
Insurance coverage and disability	213	80 (37.5)	86 (40.4)	47 (22.0)
Laboratory and other tests	326	202 (61.9)	49 (15.0)	75 (23.0)
Life impact of chronic illness	458	332 (72.4)	112 (24.2)	14 (3.0)
Lifestyle measures	116	64 (55.2)	28 (24.1)	24 (20.1)
Mental health	110	76 (69.1)	27 (24.5)	7 (6.4)
Patient journey	527	384 (72.9)	91 (17.3)	52 (9.9)
Relationship issues	303	199 (65.7)	87 (28.7)	17 (5.6)
Sexual performance and sexual health	122	77 (63.1)	21 (17.2)	24 (19.7)
Symptoms	725	460 (63.4)	234 (32.3)	31 (4.3)
Treatment discussion	1187	570 (48.02)	472 (39.76)	145 (12.21)

As seen in Table 3, the original Facebook post content topic is often associated with different levels of engagement, with some content topics requiring more moderation than others. In addition, the social support provided or solicited within the context of content topics can differ greatly. For example, 72.4% (332/458) of comments where the life impact of the health condition is discussed also include asking or giving social support, whereas only 48.02% (570/1187) of comments where treatment is discussed include a social support message. However, it is notable that regardless of the content topic, there are still high levels of social support for others expressed. Even for a *dry* topic like insurance coverage, 37.5% (80/213) of the comments were an expression of some type of support.

Content topics that have the highest proportion of comments that needed moderation of some sort, not surprisingly, are topics that are the most medical and/or technically scientific (Table 3). These are comment topics that also include insurance coverage and disability issues 22.0% (47/213), laboratory and other tests 23.0% (75/326), health care provider experiences 15.3% (45/295), and treatment discussions 12.21% (145/1187). These are also content topics with the corresponding lowest percentages of social support codes. These comment topics are more factual and may not elicit the same needs to provide validation or seek empathy and understanding compared with other topics. This may reflect the perception that these topics are more centered on individual experiences, and other community members may perceive that they require less social support than other content topics.

In addition to differing across content topics, the distribution of codes falling into each of the model’s thematic categories also differs depending on the OHC from which the comments arose. As shown in Table 4, social support was highest in the RA and migraine communities and lowest in the PC and IBS communities, although it should be noted that in these groups as well, social support was reflected in 49.49% (780/1576) and 40.3% (232/576) of the comments, respectively.

**Table 4:** Comment counts and frequency distribution of model elements by community Facebook page.

Facebook page	Comment count, N	Social support comments, n (%)	Adaptive engagement comments, n (%)	Active moderation comments, n (%)
All community FB^a^ pages	5881	3405 (57.89)	1758 (29.89)	718 (12.21)
RheumatoidArthritis.net (FB)	1417	860 (60.69)	517 (36.48)	40 (2.82)
ProstateCancer.net (FB)	1576	780 (49.49)	440 (27.92)	356 (22.60)
MultipleSclerosis.net (FB)	701	377 (53.8)	296 (42.2)	28 (3.9)
Migraine.com (FB)	876	479 (54.7)	309 (35.3)	88 (10.0)
LungCancer.net (FB)	735	397 (54.0)	255 (34.7)	83 (11.3)
IrritableBowelSyndrome.net (FB)	576	232 (40.3)	199 (34.5)	145 (25.2)

^a^FB: Facebook.

## Discussion

### Principal Findings

Results from the reach and engagement data on Facebook demonstrate that people with chronic health conditions want to engage on the web, particularly using social media. Overall, the 6 OHCs reached an average of 455,375 people per week and engaged approximately 6% to 9%, which is considered a high level of engagement on a Facebook post [95]. In addition to the benefits for those who actively engage with the content, previous research has shown that Facebook support groups provide group members who are less active with an important support network in the form of emotional support, informational support, and special companionship [34].

This highlights the importance of creating web-based spaces for the dissemination of information and facilitation of discussion regarding chronic disease. For some health conditions, people may suffer in silence because of social isolation, and the stigma of the condition may mean that online communities provide the only outlet for people to find others that are like them. In addition, people with more severe symptoms, such as those with severe MS, may look to share their experiences with others and provide support to those at earlier stages in their disease journey.

Social support is demonstrated in a variety of ways, including through information sharing, expressions of empathy, and sharing encouraging statements with others [21]. The most common manner of providing social support through Facebook comments was through the sharing of knowledge and experiences unique to people living with the chronic condition (47% of comments had this code). This shared lived experience helps members of the community, or people who visit the Facebook page, feel a bond with others living with the same health condition. This type of online community can then provide an open space for sharing things that are not necessarily understood by users’ in-person confidantes.

Previous research has shown that information seeking from people with similar experiences leads to greater perceived empathy among members of OHCs [37]. Allowing space for the expression of feelings and experiences that are not well understood by people without the condition likely helps to combat the stigma faced by people with chronic health conditions in their everyday lives. Importantly, as described above, it was much less common for these OHC members to give explicit advice to others (only 8% of comments had this code). This is important to mention as one of the common criticisms or concerns about social and peer support for health conditions is the possibility of posters sharing misinformation or pushing one’s own treatment experience on others [18]. Community guidelines that discourage giving explicit medical advice, as well as active moderation that reminds people of this rule and provides for fair balance in information sharing, are important features of this model. These OHC moderation features allow for the sharing of knowledge and experience without the downside of inaccurate and potentially misleading or dangerous unqualified medical advice.

Knowing when to let community members engage in an exchange of comments without intervening as a moderator is as important as knowing when to apply specific moderation principles. Moderation sometimes involves enforcing community rules or removing comments that violate these rules. Maintaining a safe community environment that presents balanced and medically accurate information is essential to OHCs. When needed, moderators intervene to guide the conversation away from explicit giving of advice and emphasizing that all are welcome to share their experiences, but to be mindful that everyone has different experiences with certain treatments or procedures and there is most often no one right approach or answer.

Previous research has shown that moderators can serve several functions in OHCs: provide clinical expertise, suggest users talk to their physician about their specific question, point users to outside resources, provide community expertise, build rapport, and provide technical help [97]. These results add to the finding that moderation is needed more frequently when discussing scientific and technical subjects, such as labs, diagnostic tests, insurance, and disability, than when the focus is on sharing experiences such as those related to coping or relationship issues.

The skillful use of moderation helps these OHCs function smoothly and deal with any concerning issues as they arise, ensuring that the community is not endorsing misinformation or one specific treatment approach. Additionally, moderation needs may change over time as OHCs gain maturity. The frequency of codes that reflect the need for active moderation were lowest in the migraine, MS, and RA communities. These were launched by Health Union several years before the others and represent more *mature communities* from the perspective of community management. Several factors, including the length of time a community has been active, the number of active community members, and the frequency of member engagement are associated with more mature OHCs.

Analysis of the Health Union OHC adaptive engagement model in action demonstrates that the foundational elements (social support, adaptive engagement, and active moderation) are skillfully used to provide a rich and dynamic community experience for individuals with chronic health conditions. In addition, this analysis indicates that in an open community, social support can be provided and received through the use of active moderation and adaptive engagement. This standardized structure underlying the OHCs allows for adaptation to fit the specific needs of each particular community and provides a basis to facilitate the effective and beneficial exchange of social support.

Although the authors examine a proprietary OHC model, the features of this model, as outlined in this study, are part of other OHCs as well as social media apps and proprietary health apps. Thus, these findings may be extrapolated to these other platforms as well. How consumers or people living with chronic health conditions seek, find, and use information and support is important to a range of consumer health research and applications. Patterns of use and behavior identified herein can also be used as social media apps try to adapt their most notable features to better serve OHCs.

Another implication of this research could be applied to stand-alone health apps and the need for those apps to incorporate the ability to provide moderation to better mirror the experience of OHCs. Pharmaceutical and biotechnology companies interested in social media as a means of engaging communities would be well served to consider the implications of adverse events reporting and the role of active community moderators in maintaining safe spaces for engaging communities of people with chronic health conditions.

### Limitations

The Facebook analytics data and the qualitative content of the Facebook comments presented in this study are descriptive and exploratory and are not designed for formal hypothesis testing. This study was initiated to analyze the features of support and engagement that are found organically within OHCs and enhanced through active moderation. We sought to elucidate these themes and understand whether they were consistent with the constructs of the theoretical model.

We note that a central feature of the model, that it is adaptive, is also a limitation of this research design. There is no control group to validate the extent to which social support is an inherent characteristic of OHCs or the additional effect added by features that are specific to the Health Union OHC adaptive engagement model, such as paid moderators and community rules. However, the fact that a high degree of *self-moderation* is observed among community members reacting to each other’s comments in a mutually supportive manner in the more mature communities is noteworthy.

As noted in the methods section, given the desire to demonstrate the adaptability of the OHC adaptive engagement model to a social media platform, the authors intentionally only used comments from the OHC Facebook posts for this study and did not include any comments from the linked OHC native domains. As such, the findings from online Facebook communities may not be generalizable to online communities that do not have the same interaction features as this social media platform. However, like Facebook, the trend is for OHCs, in general, to incorporate both passive and active engagement features [35].

To develop a manageable and meaningful sampling frame for this study, the random selection of posts was not feasible, which poses an additional limitation. The sampling frame was driven by the need to ensure representative content types and a large enough sample of community participant comments to determine thematic relevance and redundancy.

A final limitation of this study is the lack of longitudinal data for individual community participants. This study and other research completed by the authors to date present analyses of aggregate data from OHCs, and we have replicated the model several times with success [98]. Future research will be needed to follow distinct cohorts of OHC participants over time to assess the long-term impact of community participation on health-related behaviors and outcomes.

### Conclusions

OHCs, particularly those with clear community rules and active moderation, can offer a safe and supportive environment for people living with chronic health conditions. Despite the inherent limitations of the internet and social media, OHCs are dynamic communities of people engaging in social relationships. The dynamic nature of OHCs may make them challenging to research, but it is possible to examine the structural elements of a model in action as well as explore qualitatively and quantitatively the aspects of interactions [99].

Reaching people on the web is a promising approach to facilitate the exchange of social support among people with chronic health conditions. Health care messages bombard patients on a daily or even hourly basis. Using social media to deliver a message and provide a space for people to engage with others of similar condition-specific interests is a valuable way to cut through that noise.

OHCs such as those analyzed herein allow people with chronic health conditions to learn and engage with others on their own time and at their own pace. However, it is important to design and maintain such OHCs and their associated social media sites as safe spaces, and a structured model with essential features such as those described in this study facilitates their success. The use of a well-designed model helped the OHCs studied to achieve their primary aim: to meet people where they are at with the health information and social support they seek.

Community-responsive content, or content tailored to the needs of the community, is the foundation of an OHC. However, content alone does not provide sufficient experience for an engaging OHC. It is important to provide both active and passive opportunities for people to engage with other community members. In addition, active moderation is needed to ensure a balance of views, appropriate tone, and recognition of community rules and values.

Future research is needed to identify segments of OHCs that have different needs and different patterns of interactions. A comparative analysis of these community segments may be used to help design the features of online communities that provide for more real-time interaction, engagement features, and personalized content.

Focusing on the ongoing relationship with community members, which is enhanced through active moderation and adaptive engagement, provides a community experience that is mutually supportive and results in a healthy online community that can thrive and mature. This study has practical significance as it helps to demonstrate the value of OHCs for people living with chronic health conditions and provides support for the use of an underlying structured model to guide community interactions.

## Appendix

Multimedia Appendix 1 Facebook posts linked articles.

Multimedia Appendix 2 Codebook.

## Abbreviations

CCA: combined content analysis
IBS: irritable bowel syndrome
IRR: interrater reliability
LC: lung cancer
MS: multiple sclerosis
OHC: online health community
PC: prostate cancer
RA: rheumatoid arthritis
