# Supplementary material for: Assessment of Adaptive Engagement and Support Model for People With Chronic Health Conditions in Online Health Communities: Combined Content Analysis
Source: J Med Internet Res. 2020 Jul 7;22(7):e17338. doi: 10.2196/17338 (PMC7380984; doi:10.2196/17338)
Supplement: Multimedia Appendix 1 [file jmir_v22i7e17338_app1.docx]

Multimedia Appendix 1. Facebook posts linked articles

The 39 Facebook posts analyzed in this paper were all linked to original articles on Health Union OHC websites. These articles are linked below.

| **Community website** | **Link to Articles** | **Reference** |
| --- | --- | --- |
|  |  |  |
| **Multiple Sclerosis** |  |  |
|  | <https://multiplesclerosis.net/living-with-ms/till-you-get-it/> | [54] |
|  | <https://multiplesclerosis.net/living-with-ms/30-facts-world-ms-day> | [55] |
|  | <https://multiplesclerosis.net/living-with-ms/how-social-media-helped-overcome> | [56] |
|  | <https://multiplesclerosis.net/living-with-ms/speaking-of-advocacy> | [57] |
|  | <https://multiplesclerosis.net/topic/for-caregivers-a-sixth-stage-of-grief/> | [58] |
|  | <https://multiplesclerosis.net/q-and-a/impacted-friendships-relationships/> | [59] |
|  | <https://multiplesclerosis.net/stories/i-get-tired-of-fake-fluff-sometimes> | [60] |
| **Migraine** |  |  |
|  | <https://migraine.com/q-and-a/emotional-wreck-during-after> | [61] |
|  | <https://migraine.com/living-migraine/loving-someone-chronic-pain> | [62] |
|  | <https://migraine.com/living-migraine/the-messy-wake-of-destruction> | [63] |
|  | <https://migraine.com/living-migraine/losing-time> | [64] |
|  | <https://migraine.com/living-migraine/a-note-on-prevention-and-what-not-to-say-to-someone-with-chronic-migraine> | [65] |
|  | <https://migraine.com/living-migraine/no-nagging-please> | [66] |
|  | <https://migraine.com/living-migraine/patient-perspective-migraine-symptoms> | [67] |
| **Irritable Bowel Syndrome** |  |  |
|  | <https://irritablebowelsyndrome.net/spotlight/ibs-awareness-month-2018/> | [68] |
|  | <https://irritablebowelsyndrome.net/infographic/beyond-bathroom-impact-ibs-qol/> | [69] |
|  | <https://irritablebowelsyndrome.net/video/wish-others-knew-about-ibs/> | [70] |
| **Rheumatoid Arthritis** |  |  |
|  | <https://rheumatoidarthritis.net/living/ra-impacts-much-just-bones-joints> | [71] |
|  | <https://rheumatoidarthritis.net/living/and-the-zombie-apocalypse> | [72] |
|  | <https://rheumatoidarthritis.net/living/suiting-up-office> | [73] |
|  | <https://rheumatoidarthritis.net/living/what-tell> | [74] |
|  | <https://rheumatoidarthritis.net/living/community-thoughts-wish-more-knew> | [75] |
|  | <https://rheumatoidarthritis.net/living/pain-is-like-an-onion> | [76] |
| **Lung Cancer** |  |  |
|  | <https://lungcancer.net/stories/dont-want-to-fight> | [77] |
|  | <https://lungcancer.net/living/dear-patient><https://lungcancer.net/living/stigma-kills> | [78,79] |
|  | <https://lungcancer.net/living/pressure-of-looking-fine> | [80] |
|  | <https://lungcancer.net/living/still-get-nervous-every-scan> | [81] |
|  | <https://lungcancer.net/living/protecting-love-worry> | [82] |
|  | <https://lungcancer.net/living/a-tweak-here-a-twinge-there> | [83] |
|  | <https://lungcancer.net/living/the-road-to-grateful-not-hateful> | [84] |
| **Prostate Cancer** |  |  |
|  | <https://prostatecancer.net/living-coping> | [85] |
|  | <https://prostatecancer.net/clinical/nerves-prostate-treatment-target> | [86] |
|  | <https://prostatecancer.net/living/psycho-sexual-effect/> | [87] |
|  | <https://prostatecancer.net/symptoms/pain> | [88] |
|  | <https://prostatecancer.net/living/bombs-dropped> | [89] |
|  | <https://prostatecancer.net/diagnosis/gleason-score> | [90] |
|  | <https://prostatecancer.net/living/day-learned-prostate-cancer> | [91] |
|  | <https://prostatecancer.net/treatment> | [92] |
