# Supplementary material for: Assessment of Adaptive Engagement and Support Model for People With Chronic Health Conditions in Online Health Communities: Combined Content Analysis
Source: J Med Internet Res. 2020 Jul 7;22(7):e17338. doi: 10.2196/17338 (PMC7380984; doi:10.2196/17338)
Supplement: Multimedia Appendix 2 [file jmir_v22i7e17338_app2.docx]

Appendix 2. Codebook: Coding schema, primary codes and sub-code descriptions

| **Primary Code: Social Support** | |
| --- | --- |
| **Sub-code** | **Sub-code description** |
| Sense of Belonging/Group Membership | Any language indicative of community members establishing group boundaries, membership etc. e.g. call each other “spoonies,” etc. (“aren’t you glad we have a place to share”). |
| Shared Knowledge Network | Any language showing the shared condition experience. |
| Empathy and Understanding (from fellow community members) | Any language where community members relate and express having similar experiences, agreeing and mirroring sentiment as a means of providing social support. (e.g. “Prayers for you”). |
| Giving Advice | Any language where (non-medical) advice is given to others. E.g. Information about doctors. |
| Expressing Stigma | Any language where social stigma about living with a condition/s is expressed. (e.g. For those with COPD, people might think they caused their condition by smoking). |
| Encouragement and Motivation | Any language where community members support and motivate each other in their condition journey. (e.g. “Hang on”). |
| Caregiver Perspective | Caregivers sharing their perspective and experience with the condition/s. |
| Advocacy (and other shared goals) | Community members talking about advocacy issues like awareness, better treatment, research, etc. |
| **Primary Code: Engagement** | |
| Questions from Community Members | Questions about condition. |
| Answers from Community Members | Community members answering questions and providing information to each other. |
| Conflicts/Difference of Opinion | Community members disagreeing with each other or the content of article. |
| General Comment | Any language where community members express agreement with the content, each other, or show other signs of solidarity. |
| Conversation/Discussion | Comments back and forth between 2 or more members across a comment thread. |
| **Primary Code: Moderation** | |
| Empathy and Understanding (From members of the Health Union team) | Any language where Health Union team members validate the feelings and thoughts of the community members. |
| Knowledge and Resources | Health Union team members answering questions that community members may ask, by providing information in the form of articles, etc. |
| Relate and Share Experiences | Health Union team members, specifically site specific moderators who live with the condition share their own experiences with the community members. |
| Resolve Conflicts | Health Union team members resolve conflicts among community members. |
| Maintain Community Rules | Health Union team members maintain a safe space for community members by upholding the community rules. E.g. Not allowing solicitations or promotional comments to ensure safety of community members. |
| **Comment Topics (content topic code)** |  |
| Diagnosis | Comments having information on community members’ diagnosis. |
| Labs & Tests | Comments having information on labs and tests as part of living with a chronic illness. |
| Coping with chronic illness | Language where community members talk about efforts and means taken to cope with the chronic illness. |
| Complimentary Alternative Treatment | Comments about complimentary alternative treatments community members may have tried/considering. |
| Mental Health | Comments about community members expressing the impact of chronic illness on their mental health. |
| Life Impact of Chronic Illness | Comments about community members expressing the impact of chronic illness on their mental health. |
| Caregiver Experience | Comments about community members who are caregivers sharing their perspective. |
| Healthcare Provider Experience | Comments from community members sharing their experience with HCPs. |
| Emotional Impact | Comments include: Fear, Stress, Shock, Disappointed, Hopeful, Anger, Frustration, Guilt, Nervous/Anxious, Discourages. |
| Treatment Discussion | Comments include: Treatment impact, prescription medication, Immunotherapy, Radiation, OTC medications, Chemo, New and innovative research, Biologics, Surgery. |
| Sexual performance/health | Comments about community members expressing the impact of chronic illness on their sexual health and performance. |
| Complications & Comorbidities | Comments about community members expressing the complications of their chronic conditions and comorbidities. |
| Symptoms | Comments about various symptoms or perceived symptoms of the community members chronic health condition. |
| Lifestyle Measures | Comments about community members expressing the various lifestyle changes they’ve made in order to deal with their chronic condition. |
| Insurance coverage, disability | Comments about community members expressing issues/experiences they’ve had with insurance, treatment coverage, and/or disability. |
| Relationships | Comments about community members expressing the impact of chronic illness on their relationships. |
| Patient Journey | Comments about community members sharing their patient journey with the chronic condition/s. |
